# Supplementary material for: An HSV-1-H129 amplicon tracer system for rapid and efficient monosynaptic anterograde neural circuit tracing
Source: Nat Commun. 2022 Dec 10;13:7645. doi: 10.1038/s41467-022-35355-6 (PMC9741617; doi:10.1038/s41467-022-35355-6)
Supplement: Supplementary file 1 — Supplementary Information [file 41467_2022_35355_MOESM1_ESM.pdf]

Supplementary Information for

An HSV-1-H129 amplicon tracer system for rapid and efficient monosynaptic anterograde neural circuit tracing

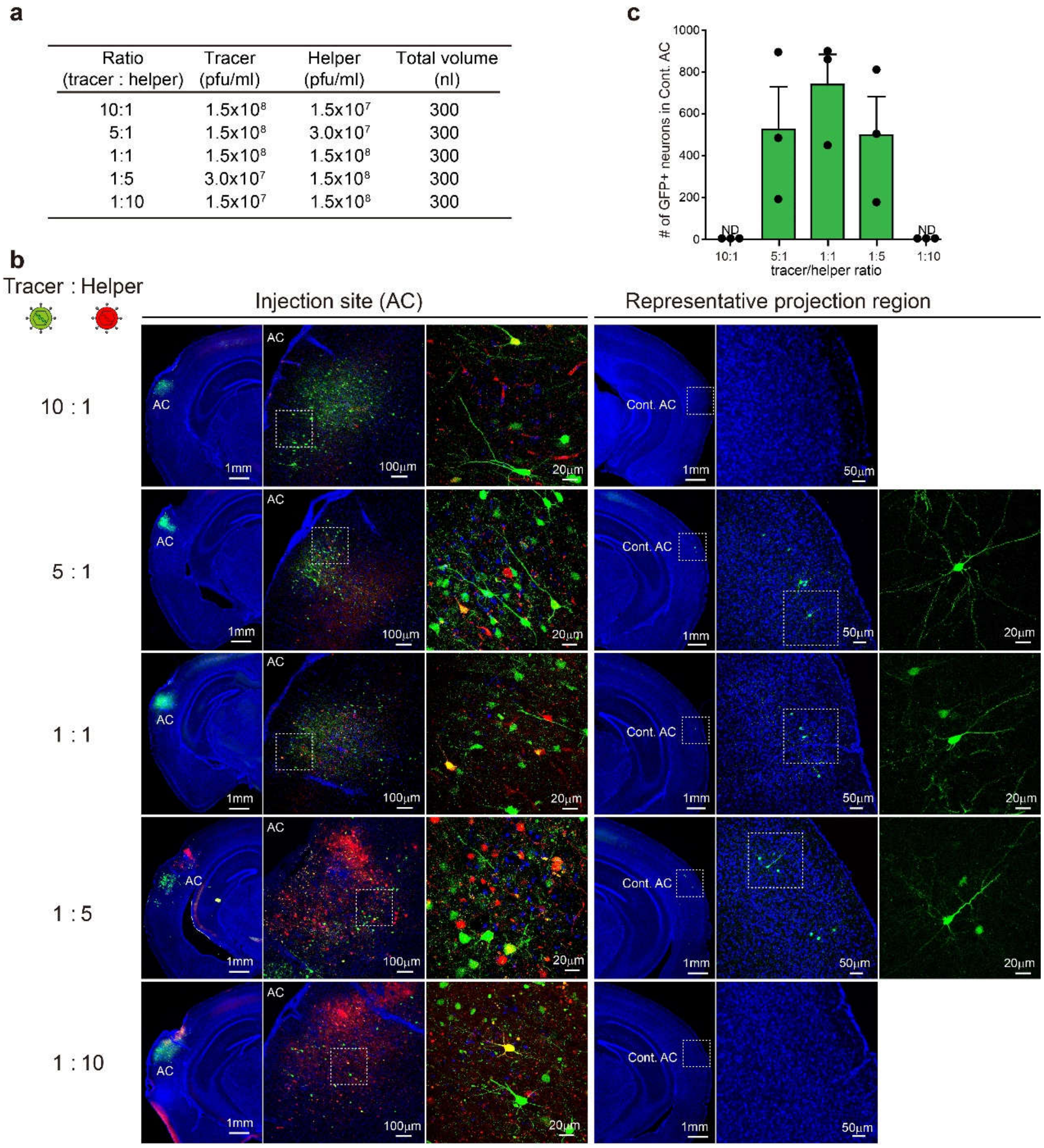

Ratio (tracer : helper)	Tracer (pfu/ml)	Helper (pfu/ml)	Total volume (nl)
10:1	1.5×10^8	1.5×10^7	300
5:1	1.5×10^8	3.0×10^7	300
1:1	1.5×10^8	1.5×10^8	300
1:5	3.0×10^7	1.5×10^8	300
1:10	1.5×10^7	1.5×10^8	300

Supplementary Fig. 1. Tracer/helper of 1:1 is the optimized ratio for anterograde monosynaptic tracing with the H129$_{Amp}$ tracer system.

Different tracer/helper ratios of H129$_{Amp}$ tracer system are tested in wildtype C57BL/6 mice. Additional H129-dTK-T2-*pac*^{Flox} helper is added to the raw tracer H129$_{Amp}$-CTG product (mixture of ~95% tracer and ~5% helper), and adjusted to the indicated titer and ratio listed in

(**a**). The mixtures, with different tracer/helper ratios, are injected into AC (AP: -2.80 mm; ML: -4.13 mm; DV: -2.38 mm) of wildtype C57BL/6 mice, and results are observed at Day 5. The representative images of the injection site (AC) and the representative AC projection site (Cont. AC) are shown (**b**), and the GFP-labeled neurons are quantified and presented as means ± SEM (10:1 0.0 ± 0.0, 5:1 525.0 ± 205.9, 1:1 740.0 ± 145.5, 1:5 498.3 ± 184.8, 1:10 0.0 ± 0.0) in Cont. AC from 3 mice (**c**). AC, auditory cortex; Cont. AC, contralateral AC. ND, not detected. Source data are provided as a Source Data file.

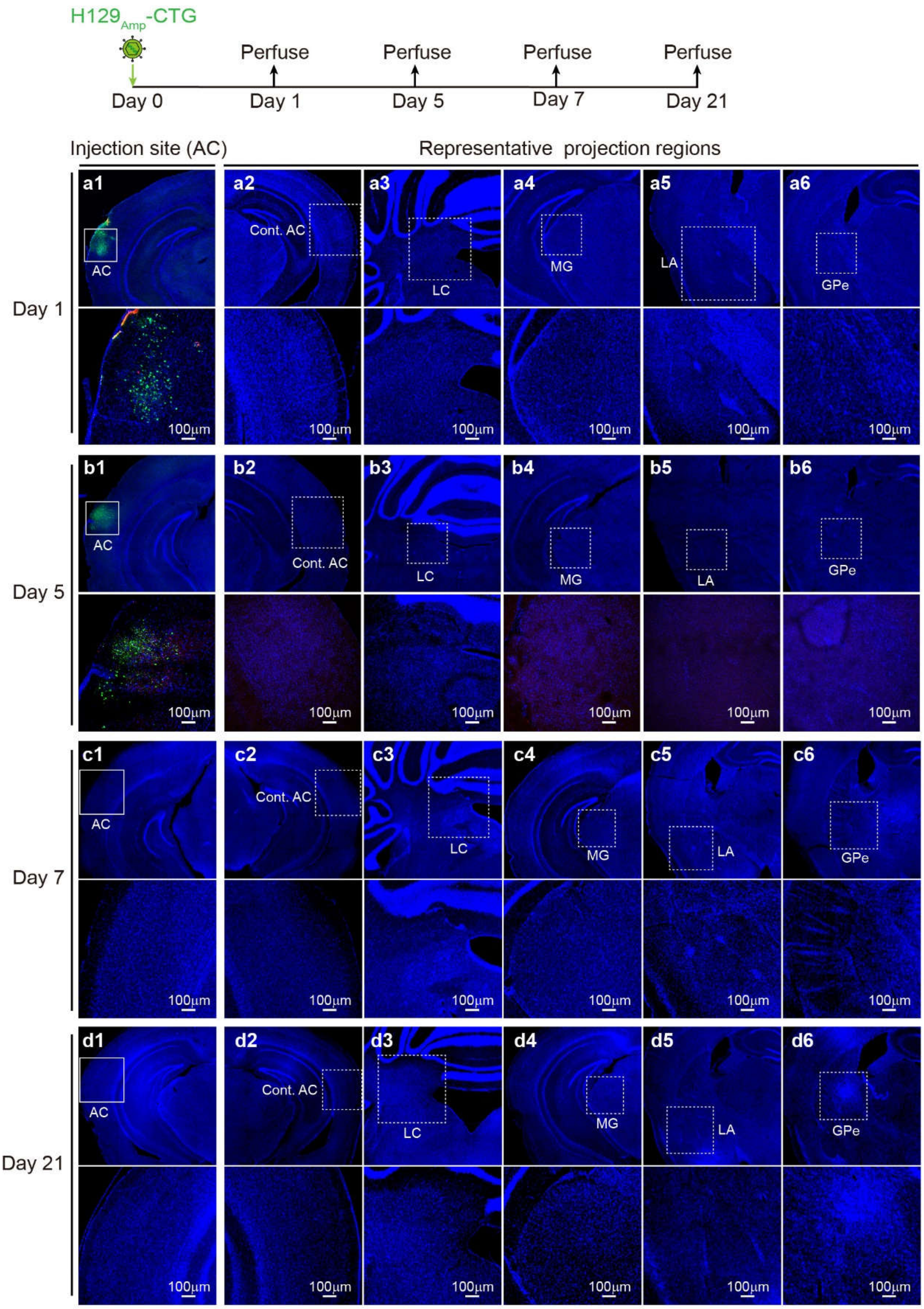

Supplementary Fig. 2. H129$_{Amp}$ tracer alone does not spread

The raw tracer product of H129$_{Amp}$-CTG (a mixture of ~95% tracer and ~5% helper) is considered as "tracer alone", since the very small amount of helper "contamination" is inevitable and irremovable in the H129$_{Amp}$ tracer product. This is titrated and adjusted to a titer of 1.5×10^{8} pfu/ml without adding additional helper. The tracer alone (H129$_{Amp}$-CTG 1.5×10^{8}

pfu/ml and 1×PBS, in 300 nl) is injected into the AC (AP: -2.80 mm; ML: -4.13 mm; DV: -2.38 mm) of wildtype C57BL/6 mice, and the brains are collected at the indicated times for imaging to examine the labeling and spread. The representative images of the injection site AC and the representative AC innervating regions are shown. Images with higher magnifications of the boxed areas are presented in the lower panels. AC, auditory cortex; Cont. AC, contralateral AC; LC, locus coeruleus; MG, medial geniculate nucleus; LA, lateral amygdala; GPe, external globus pallidus.

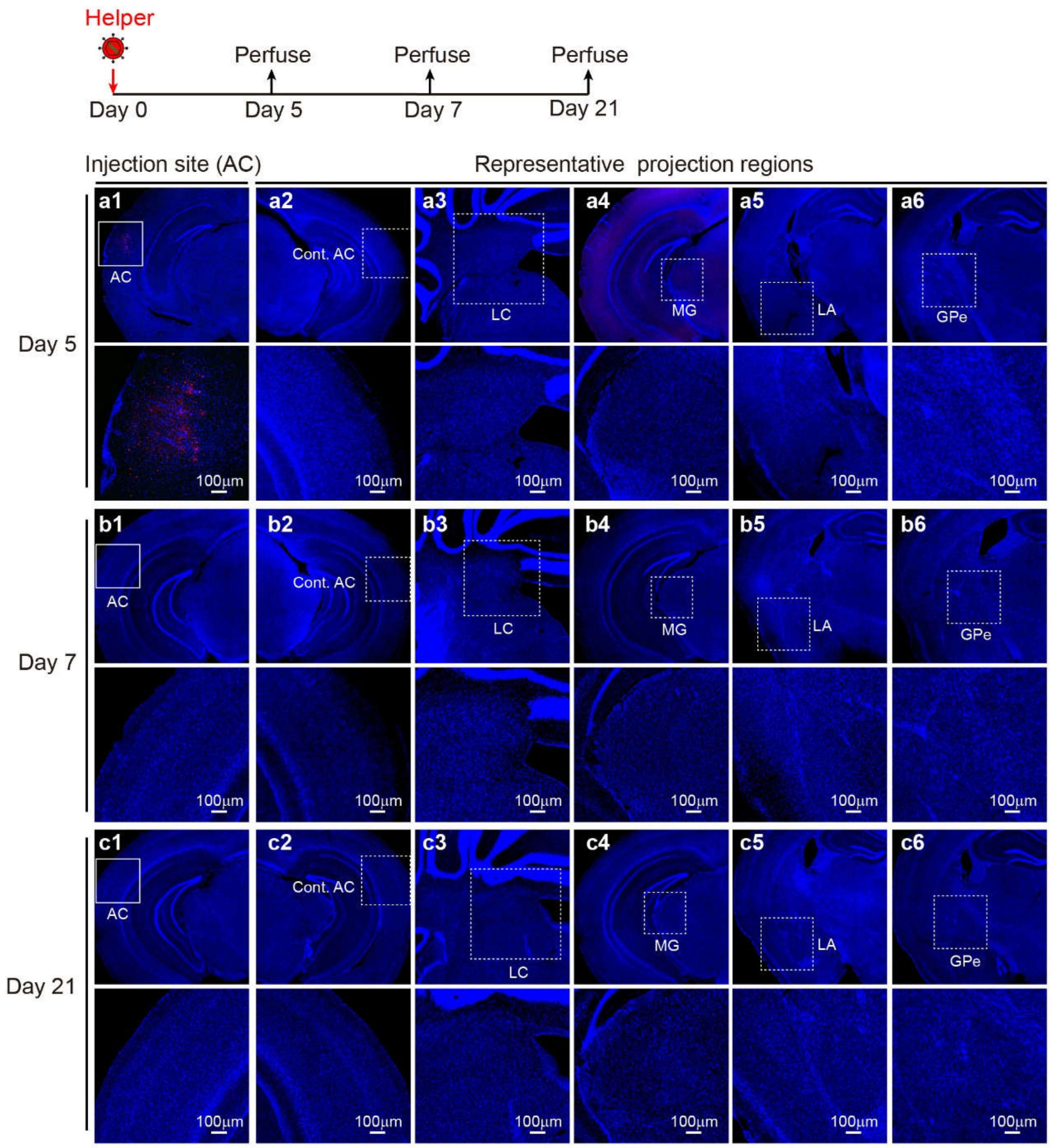

Supplementary Fig. 3. Helper alone does not spread.

Helper alone (H129-dTK-T2-*pac*^{Flox} 1.5×10^{8} pfu/ml and 1×PBS, in 300 nl) of the tracer system is injected into the AC (AP: -2.80 mm; ML: -4.13 mm; DV: -2.38 mm) of wildtype C57BL/6 mice, and the brains are collected at the indicated times for imaging to examine the labeling and spread. The representative images of the injection site AC and the representative AC innervating regions are shown. Images with higher magnifications of the boxed areas are presented in the lower panels. AC, auditory cortex; Cont. AC, contralateral AC; LC, locus coeruleus; MG, medial geniculate nucleus; LA, lateral amygdala; GPe, external globus pallidus.

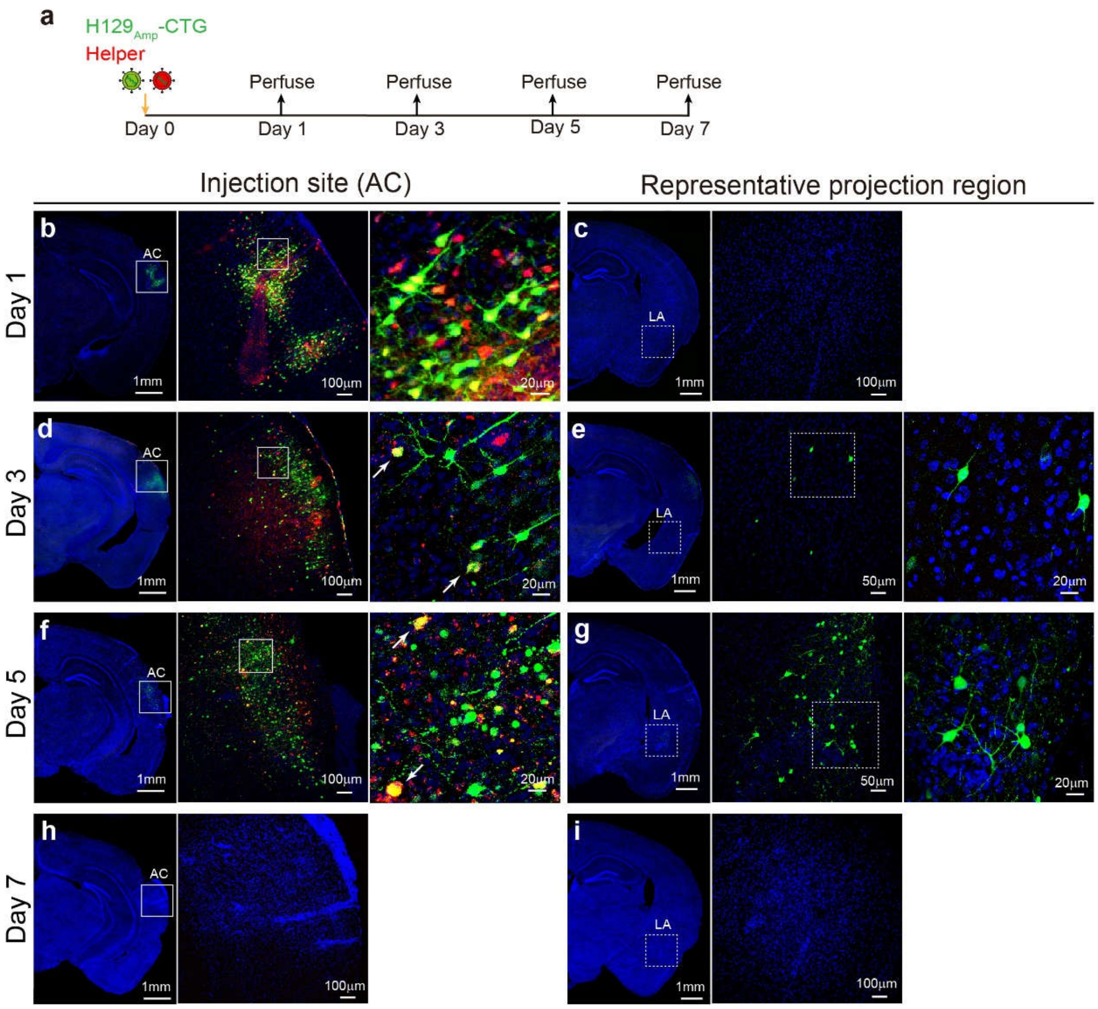

Supplementary Fig. 4. Day 5 is the optimized time for observing the postsynaptic neurons labeled with H129$_{Amp}$ tracer system.

Observation time of tracing with H129$_{Amp}$ tracer system is tested in wildtype C57BL/6 mice. The H129$_{Amp}$ tracer system (H129$_{Amp}$-CTG tracer 1.5×10^8 pfu/ml and helper 1.5×10^8 pfu/ml, in 300 nl) is injected into the auditory cortex (AC, AP: -2.80 mm; ML: +4.13 mm; DV: -2.38 mm) of wildtype C57BL/6 mice, and the brains are collected at the indicated times post-injection for imaging (**a**). The representative images of the injection site AC and the direct innervating regions, represented by LA, are shown at Day 1 (**b-c**), Day 3 (**d-e**), Day 5 (**f-g**), and Day 7 (**h-i**). Images with higher magnifications of the boxed areas are presented in the right panels. The potential starter neurons are labeled by both GFP (from H129$_{Amp}$-CTG) and tdTomato (from helper) (merged as yellow and indicated by white arrows). GFP labeled LA neurons are observed at Day 3 (**e**). The number of GFP-labeled postsynaptic neurons and GFP labeling intensity peaks at Day 5 (**g**), and then decreases and dimms at Day 7 (**i**).

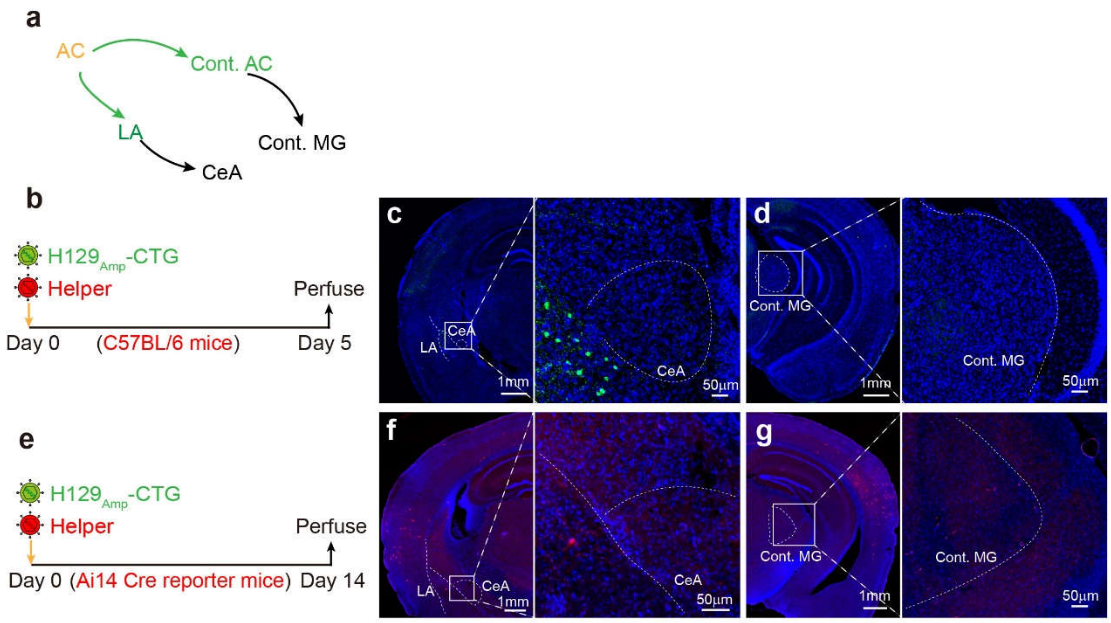

Supplementary Fig. 5. Representative 2nd-order innervating regions of AC.

The 2nd-order innervating regions of AC is tested in wildtype C57BL/6 and more sensitive Ai14 reporter mice. The simplified schematic projection pathways of AC are shown (**a**). The $H129_{Amp}$ tracer system ($H129_{Amp}$-CTG tracer 1.5×10^8 pfu/ml and helper 1.5×10^8 pfu/ml, in 300 nl) is injected into the AC (AP: -2.80 mm; ML: -4.13 mm; DV: -2.38 mm) of wildtype C57BL/6 and Ai14 mice, and the brains are collected for imaging at Day 5 and Day 14, respectively (**b and e**). The representative images of 2nd-order innervating regions of AC, represented by CeA and Cont. MG, in wildtype C57BL/6 mice (**c and d**) and Ai14 reporter mice (**f and g**) are shown. Images with higher magnifications of the boxed areas are presented in the according right panels. AC, auditory cortex; Cont. AC, contralateral AC; Cont. MG, contralateral medial geniculate nucleus; LA, lateral amygdala; CeA, central amygdaloid nucleus.

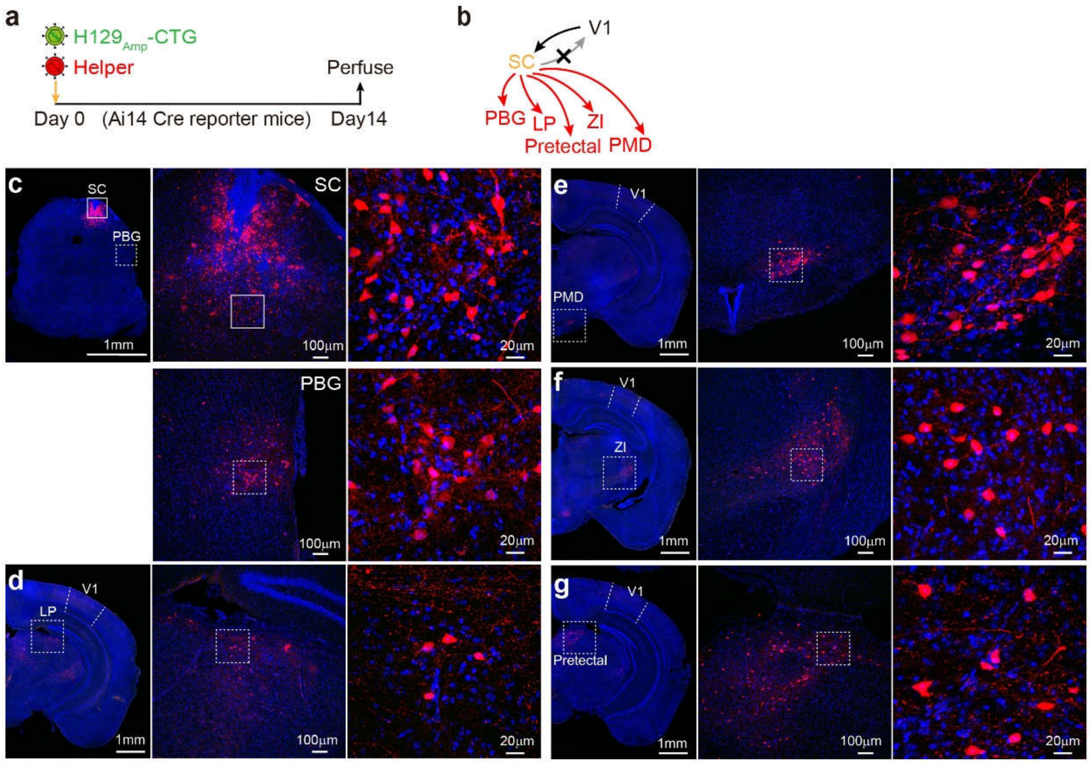

Supplementary Fig. 6. The tracing observation time window of the H129$_{Amp}$ tracer system is extended in reporter mice.

Tracing observation time of H129$_{Amp}$ tracer system is further tested in Ai14 reporter mice. The H129$_{Amp}$ tracer system (H129$_{Amp}$-CTG 1.5×10^8 pfu/ml and helper 1.5×10^8 pfu/ml, in 300 nl) is injected into the superior colliculus (SC, AP: -4.48 mm; ML: +0.6 mm; DV: -1.65 mm) of Ai14 mice, and the brains are collected at Day 14 for imaging (**a**). The simplified schematic projection pathways of SC are shown (**b**). The representative tracing results of the injection sites (**c**) and the corresponding direct downstream regions (**c-g**) are shown. The primary visual cortex (V1), a representative SC upstream region, is also displayed, and no labeled neuron soma are observed (**d-g**). Images with higher magnifications of the boxed areas are presented in the right panels. SC, superior colliculus; PBG, parabigeminal nucleus; V1, primary visual cortex; PMD, premammillary nucleus dorsal part; ZI, zona incerta; LP, lateral pulvinar nucleus of the thalamus.

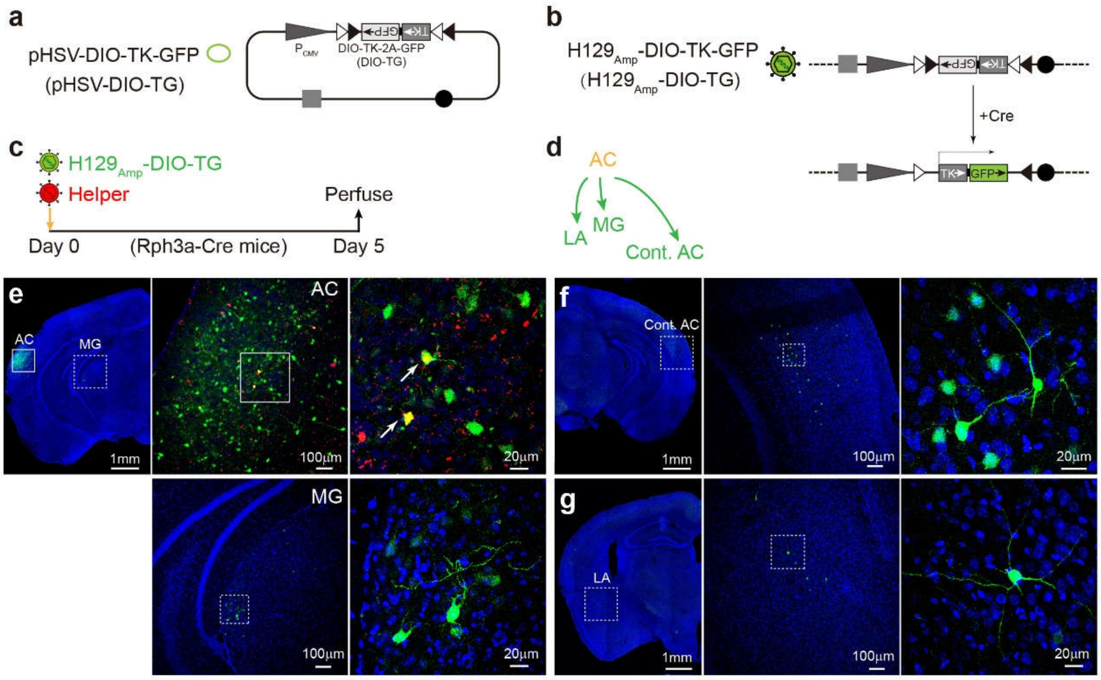

Supplementary Fig. 7. Fast-bright starter-specific monosynaptic tracing with H129$_{\mathrm{Amp}}$-tracer system.

Starter-specific tracing is tested with a modified H129$_{\mathrm{Amp}}$ tracer, H129$_{\mathrm{Amp}}$-DIO-TG, in Rph3a-Cre mice. Based on the pHSV backbone, a Cre-dependent expression cassette of TK and GFP was inserted to generate amplicon plasmid pHSV-DIO-TK-GFP (pHSV-DIO-TG) (**a**). H129$_{\mathrm{Amp}}$-DIO-TG tracer, produced with helper assistance as described in Fig.1C, expresses TK and GFP only in the presence of Cre-recombinase (**b**). The H129$_{\mathrm{Amp}}$ tracer system (H129$_{\mathrm{Amp}}$-DIO-TG tracer 1.5×10^{8} pfu/ml and helper 1.5×10^{8} pfu/ml, in 300 nl) is injected into the AC (AP: -2.80 mm; ML: -4.13 mm; DV: -2.38 mm) of Rph3a-Cre mice, and brains are collected at Day 5 for imaging (**c**). Schema of the simplified projection pathways of the AC is displayed (**d**). The representative images of the injection site AC (**e**) and the direct innervating regions (**e-g**) are shown. The potential starter neurons labeled by both GFP and tdTomato (merged as yellow) are indicated with white arrows. Images with higher magnifications of the boxed areas are presented in the right panels. AC, auditory cortex; Cont. AC, contralateral AC; MG, medial geniculate nucleus; LA, lateral amygdala.

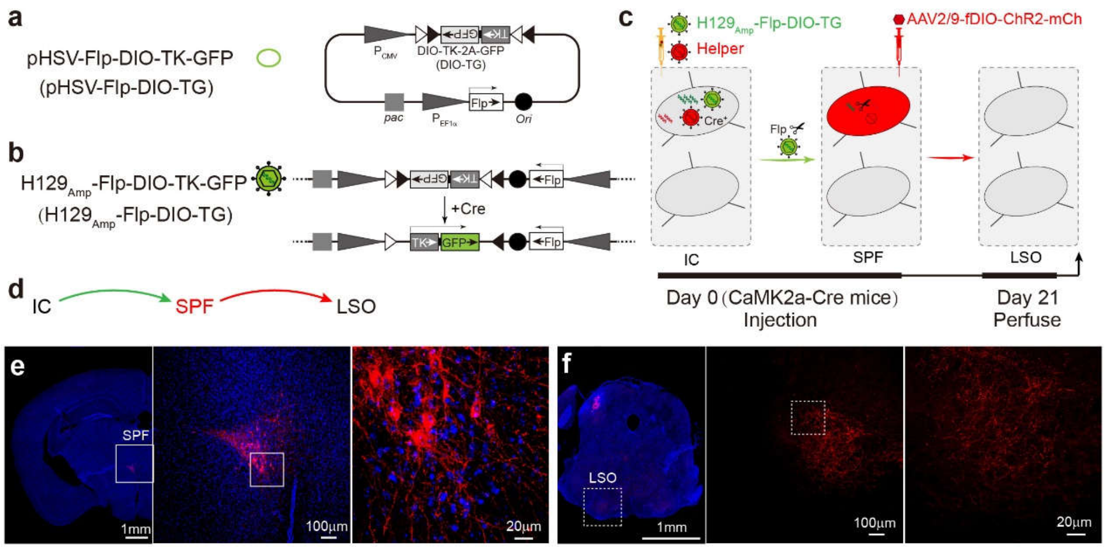

Supplementary Fig. 8. Tracing the outputs of starter-specific and input-defined neuron subpopulation with H129$_{Amp}$ tracer system.

Tracing the outputs of starter-specific and input-defined neuron subpopulation is tested with a modified H129$_{Amp}$ tracer, H129$_{Amp}$-Flp-DIO-TG, in CamK2a-Cre transgenic mice. A flippase recombinase (Flp) expression cassette was inserted into pHSV-DIO-TG to generate the amplicon plasmid pHSV-Flp-DIO-TG (**a**). The corresponding H129$_{Amp}$-Flp-DIO-TG tracer was produced as described above, which expresses Flp constantly, but expresses TK and GFP in a Cre-dependent manner (**b**). The H129$_{Amp}$ tracer system (H129$_{Amp}$-Flp-DIO-TG 1.5×10^8 pfu/ml and helper 1.5×10^8 pfu/ml, in 300 nl) is injected into the inferior colliculus (IC, AP: -4.96 mm; ML: -1.16 mm; DV: -1.75 mm). Simultaneously, AAV2/9-fDIO-ChR2-mCh (3.0×10^{12} vg/ml, 100 nl) is injected into the subparafascicular thalamic nucleus (SPF, AP: -2.30 mm; ML: -0.38 mm; DV: -4.00 mm). The brains are collected at Day 21 for imaging (**c**). As shown in the simplified schematic projection pathways, certain excitatory IC neurons innervate SPF neurons, which further project to lateral superior olive (LSO) (**d**). Representative images of soma and projection fibers of the input-defined SPF neurons (innervated by CamK2a neurons in IC) (**e**) and the representative output target region LSO (**f**) are shown. Images with higher magnifications of the boxed areas are presented in the right panels.

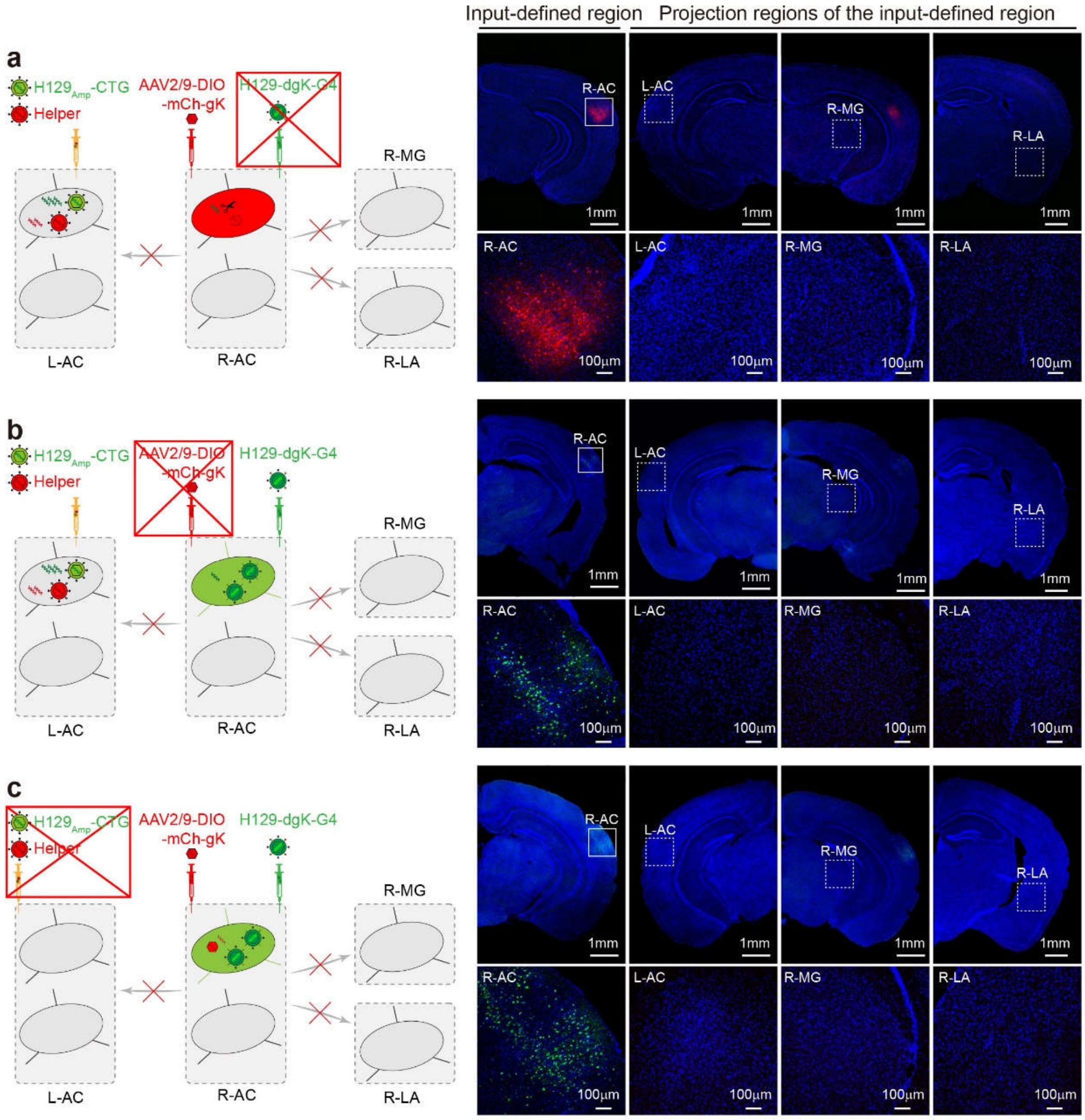

Supplementary Fig. 9. Anterograde monosynaptic tracing for input-defined postsynaptic neuron requires all components.

The $H129_{Amp}$ tracer system, AAV2/9-DIO-mCh-gK, and H129-dgK-G4 are intracranially injected into mouse brain as described in Fig. 4. We tested the effects of omitting any single component on labeling (**a-c, left panels**). The representative images of input-defined region R-AC (a target of L-AC) and its direct projection regions (the 3rd-order, targets of R-AC) are shown in the right panels. Without H129-dgK-G4 (**a**), AAV2/9-DIO-mCh-gK (**b**), or $H129_{Amp}$-CTG tracer system (**c**), no GFP signal is observed in the 3rd-order nuclei, represented by L-AC, R-LA, and R-MG. Images with higher magnifications of the boxed areas are presented in the lower panels. These results indicate that anterograde monosynaptic tracing of the input-defined postsynaptic neurons requires all components.

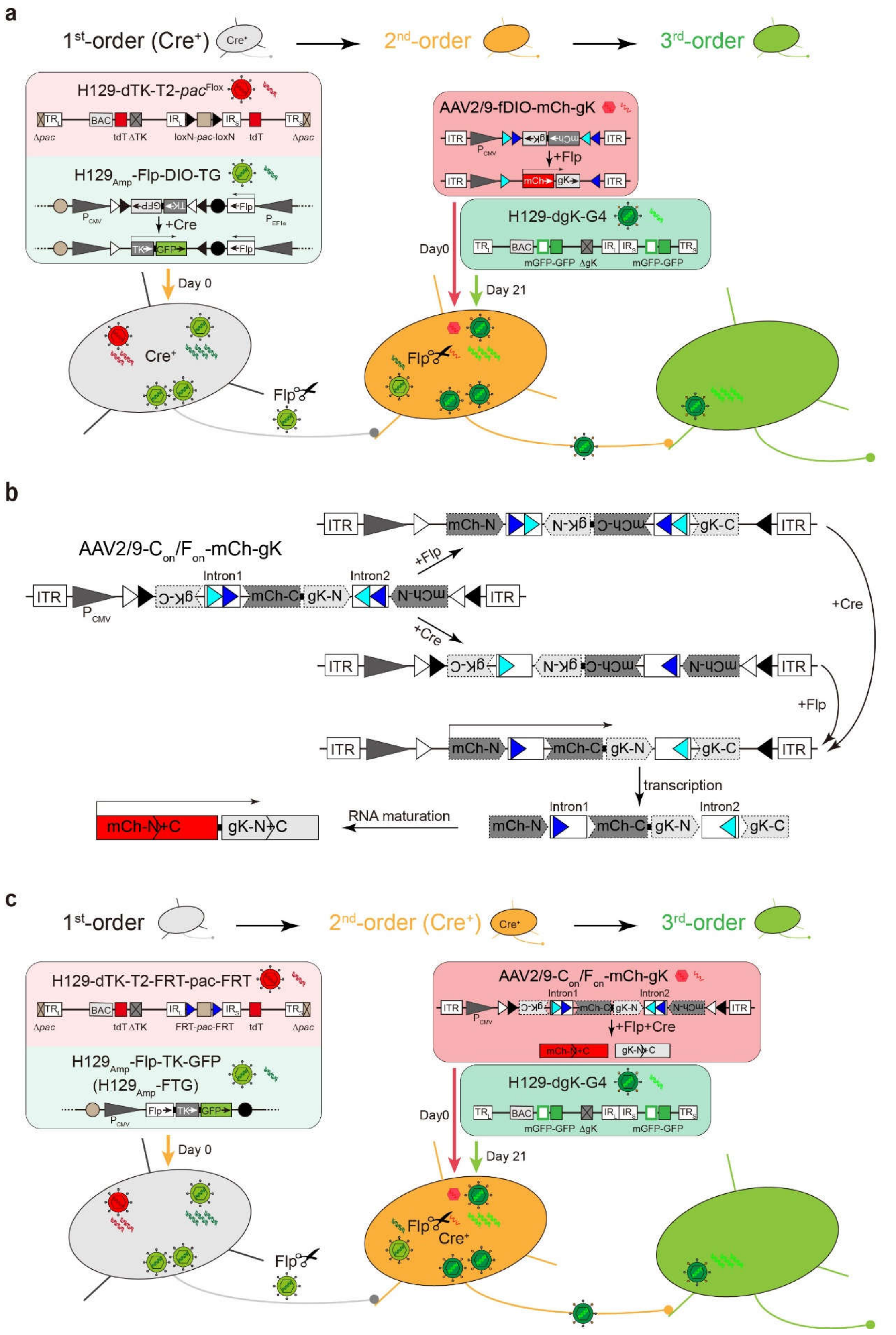

Supplementary Fig. 10. Potential H129$_{Amp}$ tracer systems and strategies for Cre-specific input-defined postsynaptic neuron' anterograde monosynaptic tracing.

Scheme of a strategy for Cre-specific input-defined postsynaptic neuron' anterograde monosynaptic tracing. (**a**) The H129$_{Amp}$ tracer system (H129$_{Amp}$-Flp-DIO-TG and H129-dTK-

T2-pac^{Flox}) is injected into the brain region of interest (1st-order) of Cre-transgenic mice, and AAV2/9-fDIO-mCh-gK is simultaneously injected into the input-defined region (2nd-order); then at Day 21, H129-dgK-G4 is injected into the 2nd-order region. In theory, $H129_{Amp}$-Flp-DIO-TG tracer propagates with helper's assistance in the Cre^{+} neurons at the 1st-order (starter), and transmits through the first synapse to 2nd-order neurons. There, the transmitted $H129_{Amp}$-Flp-DIO-TG expresses Flp-recombinase and initiates AAV2/9-fDIO-mCh-gK to express mCherry and gK, that in turn labels the neurons and supports H129-dgK-G4 propagation, respectively. The newly produced H129-dgK-G4 then transmits monosynaptically further down to the 3rd-order neurons, and labels them with GFP. (**b**) Schematics illustration of Cre- and Flp-double-dependent expression (C_{on}/F_{on}) of AAV2/9-C_{on}/F_{on}-mCh-gK. (**c**) Scheme of a strategy for the input defined- and Cre-dependent 2nd-order neuron monosynaptic tracing. The $H129_{Amp}$ tracer system, composed of $H129_{Amp}$-Flp-TK-GFP ($H129_{Amp}$-FTG) tracer and H129-dTK-T2-FRT-pac-FRT helper, is injected into the 1st-order and AAV2/9-C_{on}/F_{on}-mCh-gK is simultaneously injected into the 2nd-order of Cre-transgenic mice. H129-dgK-G4 is injected into the 2nd-order region at Day 21. In theory, $H129_{Amp}$-FTG propagates with helper's assistance in the 1st-order neurons, and transmits monosynaptically to the 2nd-order neurons, and expresses Flp there. Only in the Cre^{+} neurons, Flp expressed by $H129_{Amp}$-FTG and Cre expressed by the cells together initiate AAV2/9-C_{on}/F_{on}-mCh-gK expressing mCherry and gK, which further labels neurons and supports H129-dgK-G4 propagation, respectively. The newly produced H129-dgK-G4 finally transmits monosynaptically to the 3rd-order neurons, and labels them with GFP.

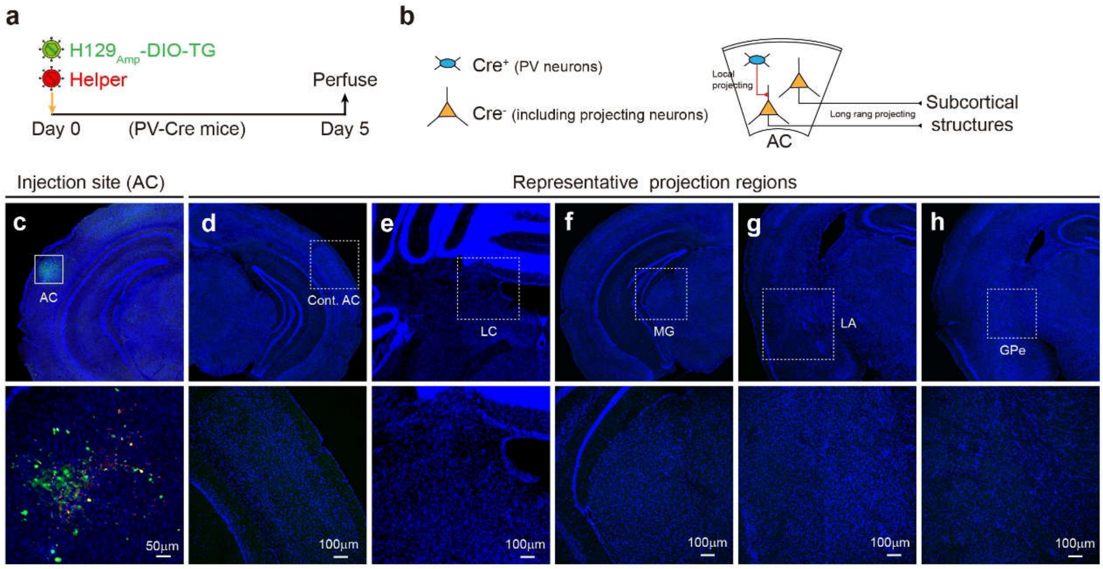

Supplementary Fig. 11. The tracer does not transmit from the Cre$^-$ neurons in starter-specific monosynaptic tracing with H129$_{Amp}$ tracer system.

In theory, it is possible that H129$_{Amp}$-DIO-TG tracer might transmit from a Cre negative (Cre$^-$) neuron, which is infected by helper and the recombined tracer (lost Cre-dependency) transmitted from a nearby Cre positive (Cre$^+$) neuron. Potential nonspecific transmission is tested in PV-Cre mice, which express Cre recombinase in parvalbumin (PV) interneurons. H129$_{Amp}$-DIO-TG tracer system (H129$_{Amp}$-DIO-TG tracer 1.5×10^8 pfu/ml and helper 1.5×10^8 pfu/ml, in 300 nl) is injected into the AC (AP: -2.80 mm; ML: -4.13 mm; DV: -2.38 mm) of PV-Cre mice, and the results are examined at Day 5 (**a**). PV neurons in AC make only local cortical connections, but some other Cre$^-$ neurons nearby may form long-range projection (**b**). The green (GFP) labeled neurons are only observed at the injection site, but not detected in any other examined brain regions. The representative images of the injection site (**c**) and representative AC-projection regions (**d-h**) are shown, and the boxed areas are displayed with higher magnifications in the lower panels.
