# Supplementary material for: An HSV-1-H129 amplicon tracer system for rapid and efficient monosynaptic anterograde neural circuit tracing
Source: Nat Commun. 2022 Dec 10;13:7645. doi: 10.1038/s41467-022-35355-6 (PMC9741617; doi:10.1038/s41467-022-35355-6)
Supplement: Supplementary file 3 — Reporting Summary [file 41467_2022_35355_MOESM3_ESM.pdf]

nature portfolio

Corresponding author(s): Min-Hua Luo

Last updated by author(s): Nov 21, 2022

Reporting Summary

Nature Portfolio wishes to improve the reproducibility of the work that we publish. This form provides structure for consistency and transparency in reporting. For further information on Nature Portfolio policies, see our Editorial Policies and the Editorial Policy Checklist.

Statistics

For all statistical analyses, confirm that the following items are present in the figure legend, table legend, main text, or Methods section.

n/a	Confirmed	
☐	☒	The exact sample size (*n*) for each experimental group/condition, given as a discrete number and unit of measurement
☐	☒	A statement on whether measurements were taken from distinct samples or whether the same sample was measured repeatedly
☒	☐	The statistical test(s) used AND whether they are one- or two-sided<br>*Only common tests should be described solely by name; describe more complex techniques in the Methods section.*
☐	☒	A description of all covariates tested
☒	☐	A description of any assumptions or corrections, such as tests of normality and adjustment for multiple comparisons
☐	☒	A full description of the statistical parameters including central tendency (e.g. means) or other basic estimates (e.g. regression coefficient) AND variation (e.g. standard deviation) or associated estimates of uncertainty (e.g. confidence intervals)
☒	☐	For null hypothesis testing, the test statistic (e.g. *F*, *t*, *r*) with confidence intervals, effect sizes, degrees of freedom and *P* value noted<br>*Give P values as exact values whenever suitable.*
☒	☐	For Bayesian analysis, information on the choice of priors and Markov chain Monte Carlo settings
☒	☐	For hierarchical and complex designs, identification of the appropriate level for tests and full reporting of outcomes
☒	☐	Estimates of effect sizes (e.g. Cohen's *d*, Pearson's *r*), indicating how they were calculated

Our web collection on statistics for biologists contains articles on many of the points above.

Software and code

Policy information about availability of computer code

Data collection	Brain section images were acquired using Nikon’s A1R MP+ confocal microscope equipped with a fast high-resolution galvanometer scanner. Optogenetics and electrophysiological assays was conducted using Multiclamp 700B amplifiers.
Data analysis	Brain section images were analyzed using Nikon’s A1R MP+ confocal microscope. Optogenetics and electrophysiological data were analyzed using pCLAMP 10.3 software. GraphPad Prism 9 was used to perform statistical analyses.

For manuscripts utilizing custom algorithms or software that are central to the research but not yet described in published literature, software must be made available to editors and reviewers. We strongly encourage code deposition in a community repository (e.g. GitHub). See the Nature Portfolio guidelines for submitting code & software for further information.

Data

Policy information about availability of data

All manuscripts must include a data availability statement. This statement should provide the following information, where applicable:

- Accession codes, unique identifiers, or web links for publicly available datasets
- A description of any restrictions on data availability
- For clinical datasets or third party data, please ensure that the statement adheres to our policy

Source data are provided with this paper. The plasmids, tracers, and helpers are available from the corresponding authors upon request. The raw imaging data is available upon request from the corresponding authors.

Human research participants

Policy information about studies involving human research participants and Sex and Gender in Research.

Reporting on sex and gender	N/A
Population characteristics	N/A
Recruitment	N/A
Ethics oversight	N/A

Note that full information on the approval of the study protocol must also be provided in the manuscript.

Field-specific reporting

Please select the one below that is the best fit for your research. If you are not sure, read the appropriate sections before making your selection.

- [x] Life sciences
- [ ] Behavioural & social sciences
- [ ] Ecological, evolutionary & environmental sciences

For a reference copy of the document with all sections, see nature.com/documents/nr-reporting-summary-flat.pdf

Life sciences study design

All studies must disclose on these points even when the disclosure is negative.

Sample size	The cell samples and animal numbers were based on estimation from previous studies, including our own published studies (Lo, L., et al. Neuron, 2011 and Zeng, W. B. et al. Mol Neurodegener 2017)
Data exclusions	For anatomical tracing and behavioral experiments, individual cases that were off the target region or did not have sufficient expression were excluded.
Replication	Each experiment was performed in triplicate, and the results obtained from at least three independent experiments or animals.
Randomization	Allocation of samples and animals are random.
Blinding	We set out to run blinded experiments. However, the fluorescence in PFA fixed brain slices was so obvious between groups that the investigators were not blinded for PFA during data acquisition, but imaging data analyses were performed blind to treatment.

Reporting for specific materials, systems and methods

We require information from authors about some types of materials, experimental systems and methods used in many studies. Here, indicate whether each material, system or method listed is relevant to your study. If you are not sure if a list item applies to your research, read the appropriate section before selecting a response.

Materials & experimental systems

n/a	Involved in the study	
x		Antibodies
	x	Eukaryotic cell lines
x		Palaeontology and archaeology
	x	Animals and other organisms
x		Clinical data
x		Dual use research of concern

Methods

n/a	Involved in the study	
x		ChIP-seq
x		Flow cytometry
x		MRI-based neuroimaging

Eukaryotic cell lines

Policy information about cell lines and Sex and Gender in Research

Cell line source(s)	Vero-E6 cell (Vero, ATCC# CRL-1586) and 293T/17 cells (293T, ATCC# CRL-11268) were purchased from ATCC.
Authentication	No authentication was performed on these cell lines.
Mycoplasma contamination	Vero-E6 cell (Vero, ATCC# CRL-1586) and 293T/17 cells (293T, ATCC# CRL-11268) were tested to be mycoplasma-free.
Commonly misidentified lines (See ICLAC register)	No commonly misidentified lines were used in this study.

Animals and other research organisms

Policy information about studies involving animals; ARRIVE guidelines recommended for reporting animal research, and Sex and Gender in Research

Laboratory animals	All mice received food and water ad libitum in a 12-h light/dark cycles and were maintained under conditionals of stable temperature (23-25 °C) and consistent humidity (45-55%). All mice used were the C57BL/6 background strains of either sex at 8-12 weeks old. Wildtype C57BL/6NCr1 mice were used for anterograde monosynaptic tracing, Ai14 mice were used for extending tracing observation time window. Rph3a-Cre, Camk2a-Cre and PV-Cre were used for Cre dependent anterograde monosynaptic tracing.
Wild animals	No wild animals were used in this study.
Reporting on sex	Both sexes were randomly used in this study.
Field-collected samples	No field-collected samples were used for this study.
Ethics oversight	All experiments with mice were reviewed and approved by the Institutional Review Board and Institutional Animal Welfare Committee of Wuhan Institute of Virology, Chinese Academy of Sciences (WIVA10201502). All experiments with viruses were reviewed and approved by the Institutional Biosafety Committee, and performed in Biosafety Level 2 (BSL-2) laboratory or Animal Biosafety Level 2 (ABSL-2) facilities following the Institutionally approved standard operating procedures and biosafety guidelines.

Note that full information on the approval of the study protocol must also be provided in the manuscript.
